# Antifungal Mechanism of *Ruta graveolens* Essential Oil: A Colombian Traditional Alternative against Anthracnose Caused by *Colletotrichum gloeosporioides*

**DOI:** 10.3390/molecules29153516

**Published:** 2024-07-26

**Authors:** Yeimmy Peralta-Ruiz, Junior Bernardo Molina Hernandez, Carlos David Grande-Tovar, Annalisa Serio, Luca Valbonetti, Clemencia Chaves-López

**Affiliations:** 1 Programa de Ingeniería Agroindustrial, Facultad de Ingeniería, Universidad del Atlántico, Carrera 30 Número 8-49, Puerto Colombia 081008, Colombia; 2 Department of Bioscience and Technology for Food, Agriculture and Environment, University of Teramo, Via R. Balzarini 1, 64100 Teramo, Italy; jmolinahernandez@unite.it (J.B.M.H.); aserio@unite.it (A.S.); lvalbonetti@unite.it (L.V.); 3 Department of Agricultural and Food Sciences, University of Bologna, 47521 Cesena, Italy; 4 Grupo de Investigación de Fotoquímica y Fotobiología, Universidad del Atlántico, Carrera 30 Número 8-49, Puerto Colombia 081008, Colombia; carlosgrande@mail.uniatlantico.edu.co

**Keywords:** *Colletotrichum gloeosporioides*, *Ruta graveolens* essential oil, membrane integrity, depolarization, cellular material release, fatty acids

## Abstract

Here, we report for the first time on the mechanisms of action of the essential oil of *Ruta graveolens* (REO) against the plant pathogen *Colletotrichum gloeosporioides*. In particular, the presence of REO drastically affected the morphology of hyphae by inducing changes in the cytoplasmic membrane, such as depolarization and changes in the fatty acid profile where straight-chain fatty acids (SCFAs) increased by up to 92.1%. In addition, REO induced changes in fungal metabolism and triggered apoptosis-like responses to cell death, such as DNA fragmentation and the accumulation of reactive oxygen species (ROS). The production of essential enzymes involved in fungal metabolism, such as acid phosphatase, β-galactosidase, β-glucosidase, and N-acetyl-β-glucosaminidase, was significantly reduced in the presence of REO. In addition, *C. gloeosporioides* activated naphthol-As-BI phosphohydrolase as a mechanism of response to REO stress. The data obtained here have shown that the essential oil of *Ruta graveolens* has a strong antifungal effect on *C. gloeosporioides*. Therefore, it has the potential to be used as a surface disinfectant and as a viable replacement for fungicides commonly used to treat anthracnose in the postharvest testing phase.

## 1. Introduction

Tropical fruits constitute a comparatively new group in the global commodity trade, having emerged in the international marketplace in significance only from 1970 onwards, thanks to advances in transportation, trade agreements, and shifting consumer preferences in favor of these fruits [1]. Mango is the dominant tropical fruit produced worldwide, followed by pineapples, papaya, and avocado.

Tropical fruits are affected by different diseases such as rot of fruits, buds, and roots, lead spots, cankers, and anthracnose, which are caused by diverse pathogens [2]. In this context, anthracnose is a fungal disease caused by the *Colletrotichum* spp. fungi complex (*C. gloeosporioides*, *C. acutatum*, *C. siamense*, *C. asianum*, *C. sloanei*, *C. fructicola*, *C. tropicale*, *C. karstii*, *C. dianesei*, *C. asianum*, *C. simmondsii*, *C. fioriniae*) [3]. The infections are commonly introduced in the early fruit developmental stages. However, the fungus remains quiescent as an appressorium along with an infection peg or subcuticular hyphae until the fruit starts to ripen and the postharvest anthracnose develops, leading to significant decay losses during storage and marketing [3]. In particular, *Colletotrichum gloeosporioides* is the causal fungus of the anthracnose disease that presents serious postharvest problems in fruits such as papaya, mango, banana, and citrus [3,4,5].

In order to control anthracnose, strategies like chemical fungicides have been used to manage the disease [6]. These strategies can be effective but present problems with the chemical substances applied and the growing concern for environmental and human health. For this reason, in recent decades, interest in using biological control and thus developing natural antifungal agents has been stimulated. Among natural compounds, the use of plant essential oils (EOs) and their molecules is receiving special attention due to their antimicrobial properties, being generally recognized by the FDA as safe for human consumption [7]. Essential oils (EOs) are strong-smelling mixtures with about 20 to 60 components, including terpenes, terpenoids, coumarins, and homologs of phenylpropanoids [8]. The complexity of this phytochemical matrix of plant extracts has the added advantage that different compounds in synergy/combination can inhibit the growth of phytopathogenic fungi and thus could be used as potential fungicides for the treatment of postharvest fungal infections [9,10].

Different hypotheses about essential oil antifungal mechanisms have been proposed, such as (a) cytoplasm leakage, (b) lysis and cell death [11], (c) membrane bilayer modification [12], (d) severe lesion of the cytoplasmic membrane [13], (e) inhibition of the biosynthesis of ergosterol and disruption of the integrity of the membrane [14], and (f) breaking up the energy metabolism for the inhibition of the enzymes mitochondrial ATPase, malate dehydrogenase, and succinate dehydrogenase [15]. Other mechanisms of action are the downregulation of sporulation- and growth-related genes, oxidative stress, and lipid peroxidation [16,17]. On the other hand, the antifungal activity of EOs and their components, as well as their ability to block the production of toxins, may have different levels of efficacy depending on their structure [18].

Some results suggested that *Ruta graveolens* (REO) essential oil could represent an interesting alternative in postharvest preservation [19,20,21,22]. In Colombia, ethnobotanical studies have reported its wide use in rural areas by farmers as insecticide and fungicide, especially in organic agriculture [23,24]. Álvarez, 2006 evidenced Colombian farmers’ use of *R. graveolens* plants intercropped in banana passion fruits (*Passiflora supersect*. Tacsonia) crops to decrease the incidence of anthracnosis [25], a disease caused by *Colletotrichum gloeosporioides* that affects tropical and subtropical zones [26]. We have already published the GC-MS data characterizing the REO used in this study [21]. In particular, we reported the existence of five esters, one terpenoid, four alcohols, seven ketones, three sesquiterpenoids, two coumarins and six unidentified chemicals in addition to 21 sesquiterpenes. While terpenes and sesquiterpenes make up the majority of chemicals in essential oils, ketones account for 76% of the composition in RGEO, with 2-nonanone (23.5%) and 2-undecanone (42.6%) having the highest relative abundance.

In previous works [8,27,28], we have demonstrated that emulsions of *Ruta graveolens* essential oil with chitosan, and REO alone are effective in the inhibition of *C. gloeosporioides* in vitro and in situ on tomatoes, guava, and papaya fruit. Studies in vitro showed significant morphological changes when observed under a light microscope, such as thinning of hyphae, malformation of hyphal walls, vacuolization, complete disorganization of the interior and irregular shape, disappearance of longitudinal septa, and general wilting and damage. In addition, we observed significant conidia germination inhibition. These results suggested that REO is a promising alternative for postharvest preservation, and its empirical use by farmers in Colombia is well founded. However, limited information is available on the possible mode of action of REO against *C. gloeosporioides*, and the antifungal mechanisms are not clear. For this reason, the present study aimed to obtain further insights into the mode of antifungal activity of *Ruta graveolens* essential oil and to understand the possible mechanisms of action by evaluating changes in the fungal cytoplasmic membrane (membrane depolarization, integrity, and changes in membrane fatty acids), induction of apoptosis, and modifications in the release of organic acids and extracellular enzyme activities.

## 2. Results

According to previous studies, REO exerted antifungal activity against *C. gloeosporioides* in vitro and in situ in *Carica papaya* fruit. In particular, in vitro studies evidenced a significant mycelia diameter reduction (76%) using 8.2 µg/mL of REO after seven days of treatment. When the pathogen was inoculated in situ on papaya fruit, anthracnose incidence and severity were reduced after 9 days of storage at 25 °C by 21% and 29%, respectively, thus reducing McKinney’s decay index by 30% [27,28]. In order to better understand the mechanisms of action that drive fungal inhibition, in this work, we have evaluated the effects of REO over one hour of treatment.

### 2.1. Perturbation of Membrane Integrity by REO

**Figure 1:**
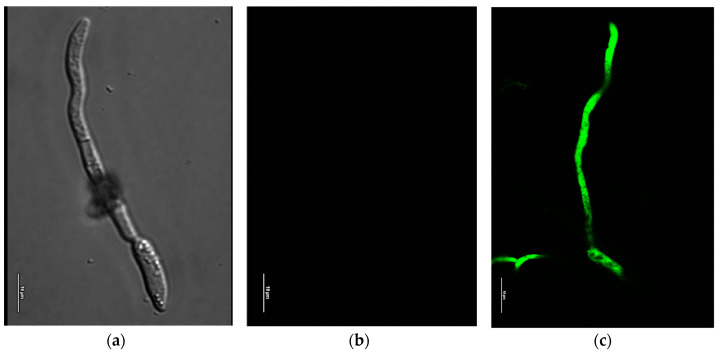
Effect of REO on membrane potential of *C. gloeosporioides*: (**a**,**b**) control sample, (**c**) sample treated with 8.2 µg/mL REO after one hour. Scale bars represent 10 µm.

Some factors influence fungal viability: membrane integrity, enzyme activity, membrane potential (ΔΨ), and nucleic acid integrity. In particular, ΔΨ is involved in cell energy production, membrane integrity, and cell physiological state [29]; thus, the structural damage of cell membranes may cause the dissipation of the membrane potential [30]. To provide evidence of whether REO was responsible for the change in ΔΨ in *C. gloeosporioides*, which is closely correlated with membrane integrity and the physiological state of fungus, we used the fluorescent green DiBAC4 dye. This label displays a high voltage sensitivity and can enter depolarized cells, where it binds to lipid-rich intracellular components bound to intracellular proteins. As shown in Figure 1, confocal microscopic observation revealed that a larger area of hyphae of *C. gloeosporioides* fluoresced in REO-treated samples than in control samples, indicating that cells were substantially depolarized as the fluorescence of DiBAC4(3) increased after 1 h of treatment. This membrane depolarization was observed in a significant part of the mycelia observed (about 80%).

**Figure 2:**
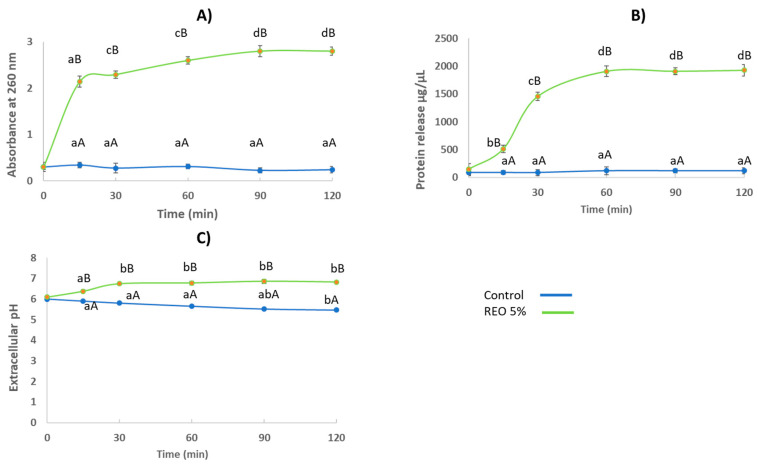
Effect of REO on the cell constituents release over time: (**A**) concentration of material released in *C. gloeosporioides* treated with REO, (**B**) concentration of released proteins, (**C**) extracellular pH. Values are the averages of the replicates for all the analyses. Error bars are ±SD of the means. Different lowercase letters in the same treatment mean significant differences among the times (*p* < 0.05). Different uppercase letters at the same time mean significant differences between treatments (*p* < 0.05).

To determine the capability of REO to disrupt the *C. gloeosporioides* cell membrane, we examined the leakage of cellular constituents and their concentration during the first 120 min after treatment (Figure 2). The DO_260nm_ of the filtrates of cells exposed to the essential oil revealed an increasing release of 260 nm-absorbing material according to the exposure time. In fact, at 15 min of exposure to REO, samples already showed a statistically significant increase in A_260_ values when compared to the control: the cellular release was nearly 5-fold greater than in untreated mycelia, indicating a substantial loss of cellular constituents in a short time. This release significantly increased (*p* < 0.05) with exposure time to REO, reaching values of nearly 8.5-fold more than the control after 120 min of exposure. The control mycelia showed that A_260_ values were slightly but not significantly reduced from 15 min to 120 min. Similarly, we also observed a significant release of proteins, which was 513.9 ± 68 μg/μL after 15 min, while in the control samples, it was 89 ± 32 μg/mL (Figure 2B); also in this case, the protein release was increased up to 60 min of exposure, reaching values of 1909.7 ± 97.3 μg/μL. These results suggest that the integrity of the cellular membrane of *C. gloeosporioides* was damaged by exposure to 8.2 µg/mL of REO, which caused intracellular leakage through the imperfect membrane. 

The release of the cellular components was accompanied by an increase in the pH of the culture medium of the mycelia exposed to the REO. As shown in Figure 2C, during the first 120 min of incubation, a significant decrease (*p* < 0.05) in the pH of the medium was observed in the control samples (from 6.0 to 5.5), which was associated with the release of organic acids. As shown (paragrahp 2.4) *C. gloeosporioides* produces a variety of organic acids during growth, such as citric acid, succinic acid, and malic acid, which are synthesized by tricarboxylic acid (TCA). In contrast, a significant (*p* < 0.05) increase in pH values from 6.1 to 6.75 was observed in the samples subjected to REO treatment during the first 30 min after treatment, followed by a moderately increasing behavior. *C. gloeosporioides* is an alkalinizing fungus via ammonia accumulation [31], and this increase in pH measured in the sample treated with REO could be associated with the faster leakage of intracellular ammonia through an imperfect membrane. In detail, ammonia is secreted by the fungus, exported as urea, and subsequently converted to NH4+ by secreted ureases. The accumulation of this fundamental compound in the immediate environment raises the pH.

### 2.2. Changes in Cell Membrane Fatty Acids

Studies have highlighted that, when microorganisms are exposed to sub-lethal stress, the cell membrane can adapt to the new environmental conditions by modifying membrane lipids, and especially the FFA profile [32]. In order to evaluate the modification of membrane lipids when *C. gloeosporioides* was exposed to REO, the FFA profile was analyzed after seven days of treatment (Table 1). 

In untreated samples, the analysis revealed a total of eleven fatty acids, which were divided into two categories: straight-chain fatty acids (SCFAs) and branched-chain fatty acids (BCFAs). The SFAs are the dominant fatty acids in the cell membrane of *C. gloeosporioides*, being 66.2% of the total fatty acids. As observed in Table 1, the profile is characterized by low presence of odd-chain FFAs and a high proportion of stearic acid (C18:0) and arachidic acid (C20:0). As regards the unsaturated FFAs, the analysis revealed only oleic acid 18:1n9C and alpha-linoleic acid 18:3n3. With exposure to REO at 28 °C, the proportion of SCFAs in the mycelia increased up to 92.1% after seven days. The proportion of odd-chain FFAs was significantly increased; notably, the undecanoic acid (C11:0) increased about 30-fold compared to the control. In addition, we detected two new odd-chain FFAs, tridecanoic acid (C13:0) and heptadecanoic acid (C17:0), which were present in low proportion. Moreover, increases in the short-chain FFAs caproic acid (C6:0) (3.7-fold) caprylic acid (C8:0) (2.5-fold), and in trans-isomers of C18:1n9 and C18:2n6 were detected. It is well known that modifications in the fatty acyl chains of membrane lipids are crucial for maintaining a level of fluidity that is consistent with membrane integrity and functionality, and are one of the tactics employed by the microorganism to adapt to changes in its environment. Moreover, the relevant accumulation of short-chain fatty acids in treated cells suggested an inhibition of the fatty acid elongation process, while a decline in overall fatty acid chain length indicates more fluidic biophysical properties for cell membranes. According to [33], reducing the chain length of fatty acids has the same effect as adding double bonds: it promotes disorder and may allow the fluid membrane necessary for healthy cell activity to exist at lower temperatures.

### 2.3. Nuclear Condensations and ROS Generation

**Figure 3:**
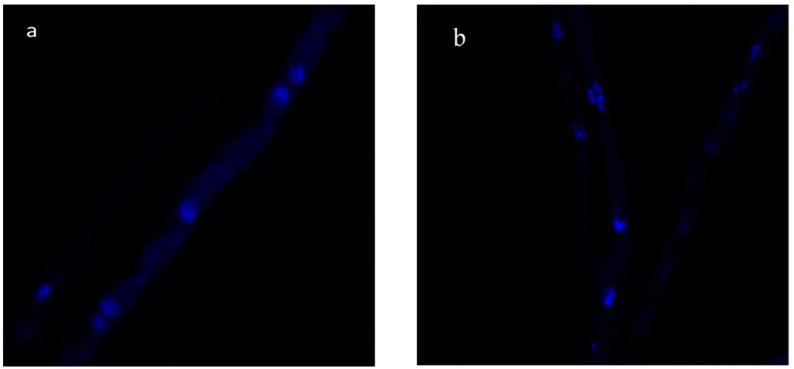
*C. gloeosporioides* (**a**) control, (**b**) treated with REO. The germinated conidium after one hour of REO treatment was fixed and stained with Hoechst 33258. The nuclei are fluorescently stained in blue. Scale bars, 10 mm.

Chromatin condensation is one of the first morphological changes associated with apoptosis; this can be easily detected by Hoechst 33258 dye, which offers an initial indication for the occurrence of cell death [34]. Thus, growing hyphae were exposed to REO to determine if its antifungal effects triggered typical apoptotic indicators. Figure 3 shows the untreated and REO-treated *C. gloeosporioides* hyphae; as evidenced, nuclei were uniformly dispersed throughout the untreated hyphae (Figure 3a), while significant nuclear condensation and DNA fragmentation on hyphae treated with REO were observed (Figure 3b). However, the results indicated that condensed nuclei were higher in hyphae exposed to REO (78%) than in control hyphae.

**Figure 4:**
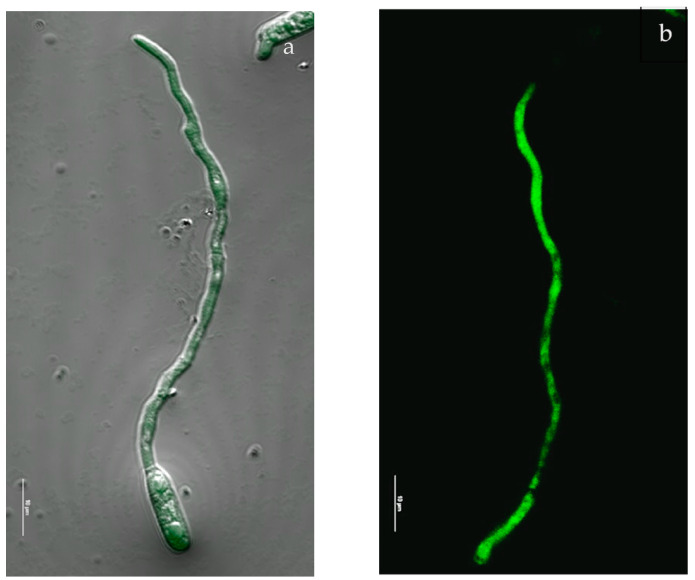
Effect of REO in ROS generation in *Colletotrichum gloeosporioides*: (**a**) control sample, (**b**) sample exposed to REO. Scale bar represents 10 mm.

It is well known that besides DNA fragmentation, ROS generation is another cause of cell apoptosis. Therefore, we studied if REO promoted ROS production [35]. Figure 4 shows *C. gloeosporioides* treated with REO and the untreated control. After six hours of incubation, the cells were stained by H_2_DCFDA dye, an indicator of ROS formation. Figure 4a shows that a very weak fluorescence was detected in control samples. On the contrary, in the treated sample, a significant increase in green fluorescence, proportionate to the amount of ROS generated, was distributed along the cytoplasm and the cell membrane, indicating a high ROS accumulation.

### 2.4. Metabolic Changes in the Cells

The metabolism response elicited by REO stress was determined by extracting and measuring organic acids and enzymatic activities from the culture medium after one hour and 3, 5, and 7 days of exposure to REO. The secretion of organic acids in fungi is determined by genes and affected by the environmental conditions of the culture [36]. Some organic acids with low molecular weight, such as malic, formic, succinic, pyruvic, and citric acids, were secreted by *C. gloeosporioides* in control and REO-treated mycelia (8.2 µg/mL) (Table 2). 

In general, exposure to REO leads to a significant increase in all the organic acids studied here. In particular, after one hour of REO exposure, citric acid (32.4%), followed by formic (26.2%), pyruvic (25.7%), succinic (25.3%), and malic acids (18.7%), was more abundant than in control samples. It is worthy of mentioning that with an increase in the exposure time, we observed a substantial increase only for citric, malic, and succinic acids, which reached values up to 95.3 mg g^−1^ dry weight mycelium (DMW) after 7 days, and 30.3 and 67.3 mg g^−1^ DMW after 5 d, respectively. Lactic and acetic acids were not detected in the culture medium during the experimental time. It is important to note that both the treated and control samples showed a significant decrease in the organic acid content during the experimental time. This suggests that the hyphae absorbed as much carbon as the growth rate permitted and then modified the activity of the catabolic metabolic pathways to satisfy the varied needs of the different nutrient limitations.

**Figure 5:**
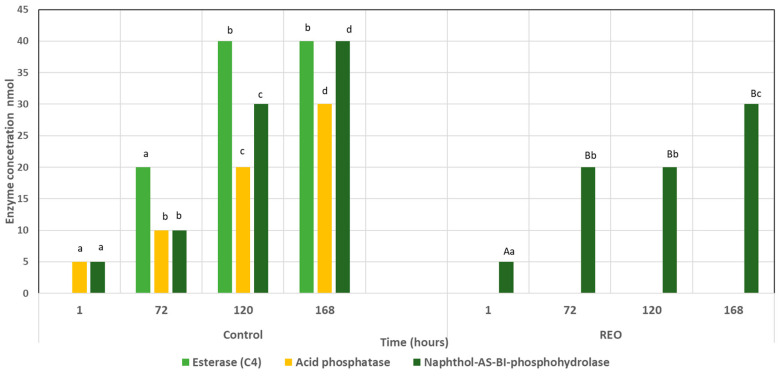
Enzymatic production by *C. gloeosporioides* in Czapek broth treated with REO (8.2 µg/mL). Different lowercase letters in the column mean a significant difference during time of incubation (*p* < 0.05), and different uppercase letters in the column mean a significant difference between the treatments.

The extracellular enzymatic activity of *C. gloeosporioides* untreated and treated with REO was studied because of the importance of the activity changes related to the responses of the microorganism to new environmental or toxicological conditions [37]. In the untreated samples, we only detected activity of Naphthol-AS-BI-phosphohydrolase and acid phosphatase in the semiquantitative test API, on a total of 19 enzymes. In fact, we evidenced the presence of acid phosphatase after one hour of incubation, while starting from 72 h of incubation, esterase (C4) was also detected (Figure 5). All the enzymes mentioned above increased with time. However, we only detected activity of Naphthol-AS-BI-phosphohydrolase in samples treated with REO (Figure 5).

## 3. Discussion

Essential oils are a complex mixture of volatile substances; plant biosynthesis of these compounds is generally associated with defense responses to pathogen attacks, physiological stresses, and ecological factors [38]. In addition, they are well known for their significant antifungal, antimicrobial, antiviral, and insecticidal properties [39]. Essential oils have received significant attention as novel antifungal agents, and this is the case for REO, which is extracted by distillation from *Ruta graveolens*. It has shown significant antifungal activity against *F. oxysporum*, *A. alternaria*, and *A. flavus* at concentrations < 163 µg/mL, and the antifungal effect improves as the concentration of the essential oil increases. Moreover, the suggested inhibition effect could be attributed to the high content of ketones and some monoterpenes and alcohols that are contained in the essential oil [21]. In fact, in REO, ketones are the predominant compounds (76%), including 2-nonanone (23.5%) and 2-undecanone (42.6%). In previous work [28], we demonstrated that among the most abundant REO constituents, 2-Nonanol showed the strongest antifungal activity followed by 2-Undecanone, Benzyl acetate, 2-Nonanone, 2-Tridecanone, and 2-Dodecanone.

At present, the mechanism of the antifungal activity of EOs has not been precisely defined; however, some authors suggest that they cause significant membrane damage due to the destruction of the membrane integrity [40,41]. It is well known that the cell membrane plays a vital function in maintaining a homeostatic environment through exchanging materials, regulating metabolism, controlling turgor, pressure, and motility, and transferring energy and information in the cell [42]. When microorganisms are exposed to external agents that compromise the cell membrane, this event may cause the dissipation of the membrane electrical potential, the disruption of the lipid bilayer, and the formation of pores [40].

In our experiments, when *C. gloeosporioides* was exposed to REO, an early structural perturbation of the cellular membrane was detected. This effect led to a depolarization of the cell membrane after one hour of interaction with the REO treatment. It is worth noting that altered depolarization of the cell membrane does not always lead to cell death, but that this may depend on the degree of alteration or whether the functionality of the cells is impaired [36]. In addition, we detected a significant release of intracellular constituents, which is generally considered an indicator of gross and irreversible damage to the cell membrane [43]. This high release was followed by a significant reduction in the mycelia dry weight (Figure S1), suggesting a solid compromise of the cell membrane. Thus, the change in the fungal membrane potential, release of cellular constituents, and extracellular pH observed in our study support the hypothesis that the accumulation of REO compounds in the cell membrane can induce immediate loss of integrity and increased permeability to ions, which could be responsible for the antifungal activity of this essential oil. Peralta-Ruiz et al. (2020) reported that, among the most abundant components of rue essential oil, 2-Nonanol showed the highest antifungal activity with an MIC value of only 0.39 µL/mL, and led to morphological abnormalities in fungal mycelia. In fact, coiled hyphae appeared more flaccid in treated mycelia, suggesting damage to the cellular membrane integrity. In this context, 2-Nonanol also reduced the ergosterol content in *P. expansum* to 44.77% [44]. 

Some researchers have shown similar results using other essential oils; for example, treatments with the EO of *Artemisia absinthium* L., which contains the most common chemical components α-Fenchene, Sabinene, and β-Thujene, and with the EO of *Mentha x piperita*, whose antifungal activity is mainly due to menthol and/or menthone, against *Candida parapsilosis,* led to a depolarization of the cell membrane [45,46]. Both the oils of *Ocimum basilicum* and *Cymbopogon citratus* and their main components (eugenol, citral a + b) were responsible for changing the selective permeability of the *Colletotrichum musae* cell membrane [47]. Similarly, *Citrus reticulata* essential oil, which is rich in citral, disrupted the integrity of the cell membrane and also caused the leakage of cell components on *Penicillium italicum* and *Penicillium digitatum* [48]. In addition, Chen et al. (2020) reported the release of soluble protein in *Fusarium oxysporum* and increased cell membrane permeability in mycelia treated with *Mentha haplocalyx* Briq essential oil, which contained D-carvone and *D*-limonene as major components [49].

Several studies have demonstrated the critical role of the cell membrane in microbial stress adaptation. It is well known that the membrane performs vital functions such as nutrient uptake and proton-motive force regulation, and it is the interface between the external environment and the cellular cytoplasm; thus, the membrane is the first target of environmental stress [50,51]. One of the principal changes, when the fungal membrane is under stress, is represented by the restriction of proton permeation, the adjustment of channel size, and changes in lipid composition [52] with the consequent modification of membrane fluidity and integrity. In this context, membrane fluidity results from several factors such as FFA chain conformations, rotational, lateral, and trans-bilayer diffusion, and resistance to shear forces [53,54]. Our work observed a significant change in the FFA profile of the *C. gloeosporioides* fungal membrane after 168 h of REO exposure. These changes were related to a pool of fatty acid enzymes that led to an increase in trans isomers, a reduction in chain length, and a general decrease in the abundance of BCFAs, suggesting a decrease in the membrane fluidity that may cause an imbalance in cell permeability and, consequently, reduce the diffusion of REO and the release of cellular constituents, opposing the environmental stress. Therefore, adapted membrane lipid compositions after treatment might have been the answer and an attempt to regulate membrane fluidity and function. In agreement with our results, Hammer et al. (2004) reported changes in fluidity and permeability in *Candida albicans* when treated with concentrations of 0.016–0.06% (*v*/*v*) of tea tree oil. Similarly, Liu et al. (2013) reported a decrease in the BCFA/SCFA ratio of the *S. cerevisiae* membrane as a stress response to D-limonene after two hours of exposure [53]. Recent studies using morphological, transcriptomic, and docking analyses evidenced that 60 μL L^−1^ of cinnamon EO downregulated the genes coding for the structural components of the cell membrane of *C. gloeosporioides* [55]. In addition, carvacrol treatment (200 μL L^−1^) promoted the production of ROS and oxidative injury, resulting in an increase in lipid peroxidation and membrane permeability of *Colletotrichum fructicola*, one of the most serious postharvest diseases in pitaya fruit [56]. On the other hand, rosemary EO was able to reduce the ergosterol content in the plasma membrane of *C. gloeosporioides* and may affect the integrity of plasma membrane [56].

On the contrary, some authors reported an increase in membrane fatty acid unsaturation following a short period of exposure to essential oils or their components, as described by Helal et al. (2006) [57], who reported an increase in BCFAs in the cellular membrane of *A. niger* after two hours of exposure to *Cymbopogon citratus* essential oil. It has been reported that BCFAs provide a high degree of fluidity and are essential to maintain a pH gradient across the cell membrane [58,59].

The surrounding environment leads to increases in physiological stress that are transduced to the activation of genes that encode for cell death and cell survival pathways [60]. Apoptosis is defined as a process of programmed cell death (PCD) defined by a set of biochemical and morphological changes, which can be activated in reaction to extrinsic and intrinsic environmental and chemical factors [34]. In this context, ROS accumulation is considered one of the major stimuli for inducing apoptosis found in eukaryotes [61] and plays an essential role as an early signal of this process [62]. In addition, ROS are a cellular sign of specific stress conditions [34] that can increase oxidative damage and reduce replicative lifespan [63]. In our work, we observed that fungal cells displayed some apoptosis markers such as nucleus condensation, DNA fragmentation, and ROS accumulation after one hour of REO exposure. These results agree with the observations of other authors [64], who reported that the exposure of *Aspergillus flavus* to clove and rosemary EOs induced significant apoptosis-like cell death, evidenced by the detection of nuclear condensation and damage of the plasma membrane. Also, Ferreira et al. (2014) reported similar results in *Saccharomyces cerevisiae* treated with *Mentha piperita* EO. Thus, the antifungal properties of REO could also be related to its potential to induce apoptosis [35].

The other two significant changes induced by the exposure of *C. gloeosporioides* to REO were the increased content of low-molecular-weight organic acids (LMWOAs) after one hour of exposure and the increased release of the enzyme alkaline phosphatase during the time of the experiment (7 days). It is well known that fungi can produce numerous LMWOAs, which play an essential role in cellular metabolic pathways as intermediates in the tricarboxylic cycle [65]. In addition, LMWOAs are specially produced when the microorganism is subjected to stress conditions, nutrient deficiency, anoxia, and environmental toxicity and can induce the motility of the microorganism through chemical signals [66]. Thus, based on our observations, a plausible speculative explanation for our results could be that the significant increase in LMWOAs after one hour of REO exposure could be related to (i) an essential requirement for energy inside the cell to repair the cellular membrane, (ii) a possible mechanism to detoxify the culture medium, or (iii) their major release due to the alteration of fungal membrane ΔΨ. which may be correlated with an enhancement in membrane permeability. To our knowledge, there are no reports related to *Colletotricum gloeosporioides* with similar results. On the other hand, the increase in Naphthol-AS-BI-phosphohydrolase (NAP), which is found in the mycelium cell wall [67] and has potential effects on the cycling of phosphorus (P) in medium, suggests that *C. gloeosporioides* activates this enzyme for cellular processes that are involved in the maintenance of cellular integrity in REO stress situations [68]. Additionally, the reduction in the enzyme’s esterase and acid phosphatase correlates well with the reduction in fungal biomass, indicating that REO toxicity interferes with the metabolic process of *C gloeosporioides*. Further transcriptomic studies will be performed to better understand the principal metabolic pathways that are affected by REO. 

## 4. Materials and Methods

### 4.1. Strain and Culture Conditions

The *Colletotrichum gloeosporioides* GGBA3 strain was used, as it possessed a high degree of pathogenesis to the papaya fruit. It was provided by the “Agricultural Bioprospecting Research Group of the Microbiological Research Laboratory” of the Faculty of Agricultural Sciences, Universidad de Sucre—Sincelejo, Colombia. It was cultured in Czapek broth and incubated at 28 °C.

### 4.2. Essential Oil

*Ruta graveolens* essential oil was purchased from Krauters (Bogotá, Colombia) and was characterized in our previous work [8]. Rue essential oil was applied at a concentration of 8.2 µg/mL, at which it demonstrated inhibition of *C. gloeosporioides* mycelial growth, as we previously reported [28].

### 4.3. Membrane Depolarization Assay

Alteration of membrane permeability was determined following the method reported by Lee and Lee (2015) with some modifications [69]. *C. gloeosporioides* spores in a 10^5^ spore/mL concentration were incubated in microscope slides with Czapek broth at 28 ± 2 °C for 16 h, followed by treatment with a sub-lethal REO concentration for one hour. To analyze membrane disturbances due to REO, the cells were treated with 20 µg/µL of bis-(1,3-dibutylbarbituric acid) trimethine oxonol [DiBAC4(3)] and incubated for 30 min in the dark. Stained *C. gloeosporioides* cells were examined under an Olympus fluorescence microscope Nikon A1R confocal imaging system (Nikon Corp., Tokyo, Japan) and controlled by the Nikon NIS Elements interface, equipped with a Plan Apo k 100 9 Oil objective (numerical aperture: 14; Refractive Index: 1515).

### 4.4. Release of Cellular Material

The release of cellular contents was determined according to the method of Tao et al. (2014b) with some modifications [48]. Mycelia of *C. gloeosporioides* in Czapek broth were centrifuged for 20 min at 6000 rpm; afterwards, the pellet was washed four times with 50 mL phosphate-buffered saline (PBS). The samples were treated with a sub-lethal REO concentration and incubated at 28 ± 2 °C under agitation at 120 rpm for 120 min. The release of cellular material was determined after 0, 30, 60, 90, and 120 min; after centrifugation, 50 mL of supernatant was used to measure the absorbance at 260 nm and the DNA concentration with a bio photometer (Eppendorf, Milan, Italy). The control sample without REO was also tested.

In addition, the protein concentration of the released constituents at 0, 30, 60, 90, and 120 min was determined by the method of Bradford (1976), with bovine serum albumin as a standard [70].

### 4.5. Measurement of Extracellular pH

The extracellular pH level was determined according to the methodology reported by Tao et al. (2014b) with minor modifications [48]. *C. gloeosporioides* spores with a concentration of 10^5^ spores/mL were incubated in 50 mL of Czapek broth at 28 ± 2 °C for two days; afterwards, the mycelia were centrifugated at 6000 rpm and washed three times with sterilized double-distilled water in order to eliminate the residues of the growth media. Then, the mycelia were resuspended in 10 mL sterilized double-distilled water, adding sub-lethal REO concentrations. The extracellular pH measurements of the samples at 0, 30, 60, 90, and 120 min were carried out using a pH meter (Mettler-Toledo, Milan, Italy); a sample without REO was used as a control.

### 4.6. Change in Membrane Fatty Acid (FFA) Composition

Lipid extraction of the mycelium with sub-lethal REO concentration for 168 h was carried out according to Folch et al. (1957) [71]. For the analysis of esterified fatty acids, 1 g of treated mycelia previously grown in Czapec broth for 2 days at 28 ± 2 °C was homogenized in 20 mL of methanol–chloroform (2:1 *v*/*v*) mixture overnight in darkness at 25 °C. Afterward, the homogenate was filtered, the supernatant was decanted, and the fungal biomass was re-extracted with 10 mL of methanol–chloroform mixture (2:1 *v*/*v*) followed by two rounds of homogenization by 3 h and filtration. After filtration, the combined supernatants were diluted with 8 mL of KCl (0.1 M) and gently agitated for phase separation in a separator funnel.

The lower chloroform phase was withdrawn and passed by anhydrous sodium sulfate to remove traces of water after being concentrated in a rotary vacuum evaporator at 40 °C. The free fatty acids were dissolved in an appropriate volume (mL) of hexane suitable for HPLC > 95% purity (Sigma-Aldrich, Milan, Italy). All the experiments were repeated two times; lipids extracted by the untreated mycelium were considered as a control. Fatty acids (100 mg) were esterified with BF3 in methanol (Supelco BF3-Methanol kit) for 3 min at 60 °C following the protocol of the producers; after that, the reaction was stopped with double-distilled water, the sample was decanted, and the upper hexane phase was withdrawn and passed by anhydrous sodium sulfate. Samples were dried with N2 and successively solubilized with 1 mL of hexane for further analysis.

The analysis of FFAs, as methyl esters, was carried out using a Carlo Erba HRGC 5160 Mega Series instrument (Carlo Erba, Cornaredo, Italy), equipped with a 30 m × 0.32 mm i.d. fused silica capillary column coated with a film of thickness 0.20 μm (Supelco, Bellefonte, PA, USA). The oven temperature was programmed for 1 min at 120 °C. The temperature was increased at a rate of 10 °C per minute to 175 °C. Later, it was increased at a rate of 2.5 °C per minute to 210 °C and then increased at a rate of 5 °C per minute to 230 °C. The detector (FID) temperature was 250° C. Helium was used as carrier gas at a pressure of 290 kPa, and peaks were identified by comparison of their retention times with appropriate FAME standards (Supelco 37 Component FAME Sigma-Aldrich) (St. Louis, MO, USA).

### 4.7. Phenotypic Changes of the DNA Characteristic of Apoptosis

To determine one of the early stages of apoptosis expressed by nuclear condensation, the assays were performed according to the method of Nguyen et al. (2019) [34]. *C. gloeosporioides* spores with a 10^5^ spore/mL concentration were incubated using the procedure described in Section 4.3. Afterwards, the samples were treated with a sub-lethal REO concentration for one hour. Then, the round slides were washed twice with PBS and stained with Hoechst 33258 solution. The staining solutions for nuclear condensation as early apoptosis were prepared using a Hoechst 33258 (Sigma-Aldrich). Stock solutions of 100 mg/mL were used to obtain a diluted working solution of 12 mg/mL; successively, 5 μL working solution was added to the samples. The different round slides were observed under the Olympus fluorescence microscope Nikon A1R confocal imaging system (Nikon Corp., Tokyo, Japan) and controlled by the Nikon NIS Elements interface, equipped with a Plan Apo k 100 9 Oil objective (numerical aperture: 14; Refractive Index: 1515) to observe the changes in the nuclei.

### 4.8. Intracellular Reactive Oxygen Species

Reactive oxygen species (ROS) production was examined according to the methodology reported by Liu et al. (2010) [61]. *C. gloeosporioides* spores with a 10^5^ spores/mL concentration were incubated in slides with Czapek broth at 28 ± 2 °C for 16 h, followed by treatment with sub-lethal REO concentration for one hour. Later, the slides were washed two times with PBS and incubated with 6.5 μL of the oxidant-sensitive probe dichlorodihydrofluorescein diacetate (H_2_DCFDA-Sigma Aldrich) stock solution (5 mM in DMSO) for 15 min. The different slides were observed under the Olympus fluorescence microscope Nikon A1R confocal imaging system (Nikon Corp., Tokyo, Japan) and controlled by the Nikon NIS Elements interface, equipped with a Plan Apo k 100 9 Oil objective (numerical aperture: 14; Refractive Index: 1515).

### 4.9. Secretion of Organic Acids under REO Treatment

After seven days of incubation of *C. gloeosporioides* with a sub-lethal REO concentration, the medium was separated and filtered with a PTFE hydrophilic Millex^®^-LCR 0.45 µm syringe filter for each sample. Then, the organic acid contents were analyzed by high-performance liquid chromatography (HPLC) using an HPLC PerkinElmer Serie 200 with autosampler and UV detector set at 210 nm. Seven standard organic acids solutions, including citric, pyruvic, malic, succinic, lactic, formic, and acetic acids, were prepared for acid identification by HPLC. A 10 µL amount of each sample was injected into the chromatographic system with an ion-exchange column compatible with organic acids (300 × 8 mm × 8 µm) (Bio-Rad, Segrate, Italy) at a temperature of 45 °C and flow of 0.4 mL/min. Sulfuric acid 4.5 mM was used as the mobile phase. Identification of compounds was carried out considering the retention times of organic acid standards. Quantification was performed by calibration curves for each organic acid (Table S1). All experiments were repeated three times.

### 4.10. Semi-Quantitative Assay for Enzymes

The mycelium was grown for three days in Czapek liquid media from a fresh fungal plate. Successively, the mycelium was treated with REO, and at regular intervals (1 h, 3, 5 and 7 days), samples of the culture media were centrifuged at 13,000 rpm for 15 min, then filtered through a sterile 0.22 μm syringe filter (VWR, West Chester, PA, USA), and immediately analyzed for enzymatic activity using API ZYM galleries (BioMérieux, Marcy L’Etoile, France). This test is a semi-quantitative assay for 19 enzymes. The test was performed according to the manufacturer’s instructions. To summarize, each well of the API-ZYM^®^ system received 65 μL of the filtrate, which was then incubated for 4 h at 30 °C in the dark. A drop of ZYM A and ZYM B was then added according to the manufacturer’s instructions. The results were determined in nano *M*ol (nmol) of the hydrolyzed substrate according to the intensity of the color reaction on a scale of 1–5, i.e., 1 = 5 nmol, 2 = 10 nmol, 3 = 20 nmol, 4 = 30 nmol, and 5 ≥ 40 nmol [37]. All untreated *C. gloeosporioides* were considered as control samples. The test was repeated twice.

### 4.11. Statistical Analyses

The data regarding released cellular content, extracellular pH, secretion of organic acids, and change in membrane fatty acids are shown as the mean ± SD and were statistically evaluated by an analysis of variance ANOVA, followed by individual comparisons using Duncan’s Multiple Range Test (*p* < 0.05)

## 5. Conclusions

In view of the growing problem of fungicide resistance, new chemicals and treatment options are constantly in demand. The data obtained here have shown that *Ruta graveolens* essential oil exhibits potent antifungal effects on *C. gloeosporioides* via membrane-disrupting mechanisms, significant apoptosis-like cell death induction, reduction in enzymatic production, and inhibition of the fatty acid elongation process. These latter reductions were attributed to the lower metabolism of the fungi exposed to REO. In addition, the presence of its main constituents, 2-nonanol and 2-undecanone, contributed to the efficacy of REO on fungal growth. As a result, it has the potential to be employed as a surface disinfectant and a viable substitute for fungicides frequently used to treat anthracnose during the postharvest test stage.

## Figures and Tables

**Table 1:** Relative percentages of free fatty acids in *C. gloeosporioides* unexposed (control) and exposed to REO (8.2 µg/mL).

Fatty Acid		Control	REO
**Straight-chain fatty acids**	6:0	3.3 ± 0.21	12.3 ± 0.21
	8:0	4.4 ± 0.21	10.8 ± 0.21
	9:0	-	-
	10:0	3.1 ± 0.00	0.6 ± 0.01
	11:0	1.8 ± 0.10	54.6 ± 0.00
	12:0	2.8 ± 0.08	2.90 ± 0.12
	13:0	-	0.80 ± 0.02
	14:0	2.0 ± 0.03	-
	15:0	-	-
	16:0	2.5 ± 0.00	0.20 ± 0.01
	17:0	-	1.10 ± 0.00
	18:0	20.2 ± 1.00	0.5 ± 0.00
	19:0	-	-
	20:0	18.7 ± 1.00	5.7 ± 0.06
	21:0	-	-
	22:0	7.4 ± 0.00	3.2 ± 0.00
**Branched-chain fatty acids**	18:1n-9C	21.6 ± 0.76	-
	18:1n-9T	-	0.6 ± 0.04
	18:2n-6T	-	3.1 ± 0.08
	18:3n-3	12.2 ± 1.28	4.2 ± 0.12
	18:3n-6	-	-
	20:1T	-	-
	20:1	-	-
	20:3n6	-	-
**Total**	saturated unsaturated	66.2 ± 2.63 b	92.1 ± 0.64 a
		33.8 ± 2.04 a	7.9 ± 0.24 b

Different letters in the column mean significant differences (*p* < 0.05).

**Table 2:** Organic acids secreted in the culture medium by *C. gloeosporioides* treated and not treated (control) with REO (8.2 µg/mL).

Time	Sample	Acid Concentrations mg g^−1^ DWM				
		Citric Acid	Pyruvic Acid	Malic Acid	Succinic Acid	Formic Acid
1 h	Control	121.6 ± 11.1 ^a^	5.27 ± 0.48 ^a^	35.6 ± 3.24 ^a^	73.5 ± 6.68 ^a^	7.34 ± 0.67 ^a^
	REO	180.0 ± 5.14 ^A^	7.10 ± 0.20 ^A^	43.8 ± 1.25 ^A^	98.4 ± 2.81 ^A^	9.94 ± 0.28 ^A^
3 days	Control	32.11 ± 4.03 ^b^	1.73 ± 0.22 ^d^	8.56 ± 1.07 ^b^	20.4 ± 2.57 ^b^	4.57 ± 0.57 ^b^
	REO	149.6 ± 7.24 ^B^	5.25 ± 0.25 ^B^	40.7 ± 1.97 ^A^	83.1 ± 4.02 ^B^	6.57 ± 0.32 ^B^
5 days	Control	18.20 ± 0.10 ^c^	2.18 ± 0.01 ^c^	4.34 ± 0.02 ^c^	14.4 ± 0.08 ^c^	3.47 ± 0.02 ^d^
	REO	108.6 ± 1.91 ^C^	3.86 ± 0.07 ^C^	30.3 ± 0.53 ^B^	67.3 ± 1.18 ^C^	6.44 ± 0.11 ^B^
7 days	Control	18.32 ± 1.99 ^c^	2.53 ± 0.27 ^b^	3.93 ± 0.43 ^c^	3.54 ± 0.39 ^d^	3.72 ± 0.40 ^c^
	REO	95.33 ± 1.21 ^D^	3.35 ± 0.04 ^D^	25.3 ± 0.32 ^C^	59.1 ± 0.75 ^D^	6.13 ± 0.08 ^C^

Different lowercase letters in the column mean a significant difference over time to the control (*p* < 0.05), and different uppercase letters in the column mean a significant difference over time to REO (8.2 µg/mL) treatment.

## Data Availability

Data are contained within the article and Supplementary Materials.

## Supplementary Materials

The following supporting information can be downloaded at https://www.mdpi.com/article/10.3390/molecules29153516/s1: Figure S1: *C. gloeosporioides* mycelia growth reduction treated with REO. Table S1. Calibration curves, coefficients of determination (R^2^), and retention time for citric, pyruvic, malic, succinic, lactic, formic, and acetic acids.

## References

[1] Altendorf S. (2017). Global Prospects for Major Tropical Fruits Short-Term Outlook, Challenges and Opportunities in a Vibrant Global Marketplace.

[2] Ploetz R.C., Ploetz R.C. (2003). Diseases of Tropical Fruit Crops.

[3] Siddiqui Y., Ali A. (2014). Colletotrichum Gloeosporioides (Anthracnose).

[4] dos Passos Braga S., Lundgren G.A., Macedo S.A., Tavares J.F., dos Santos Vieira W.A., Câmara M.P.S., de Souza E.L. (2019). Application of Coatings Formed by Chitosan and *Mentha* Essential Oils to Control Anthracnose Caused by *Colletotrichum gloesporioides* and *C. brevisporum* in Papaya (*Carica papaya* L.) Fruit. Int. J. Biol. Macromol..

[5] Lima Oliveira P.D., de Oliveira K.Á.R., dos Santos Vieira W.A., Câmara M.P.S., de Souza E.L. (2018). Control of Anthracnose Caused by *Colletotrichum* Species in Guava, Mango and Papaya Using Synergistic Combinations of Chitosan and *Cymbopogon citratus* (D.C. Ex Nees) Stapf. Essential Oil. Int. J. Food Microbiol..

[6] Saxena A., Raghuwanshi R., Gupta V.K., Singh H.B. (2016). Chilli Anthracnose: The Epidemiology and Management. Front. Microbiol..

[7] da Rocha Neto A.C., Navarro B.B., Canton L., Maraschin M., Di Piero R.M. (2019). Antifungal Activity of Palmarosa (*Cymbopogon martinii*), Tea Tree (*Melaleuca alternifolia*) and Star Anise (*Illicium verum*) Essential Oils against Penicillium Expansum and Their Mechanisms of Action. LWT.

[8] Grande Tovar C.D., Delgado-Ospina J., Navia Porras D.P., Peralta-Ruiz Y., Cordero A.P., Castro J.I., Valencia C., Noé M., Mina J.H., Chaves López C. (2019). Colletotrichum Gloesporioides Inhibition In Situ by Chitosan-Ruta Graveolens Essential Oil Coatings: Effect on Microbiological, Physicochemical, and Organoleptic Properties of Guava (*Psidium guajava* L.) during Room Temperature Storage. Biomolecules.

[9] Matrose N.A., Obikeze K., Belay Z.A., Caleb O.J. (2021). Plant Extracts and Other Natural Compounds as Alternatives for Post-Harvest Management of Fruit Fungal Pathogens: A Review. Food Biosci..

[10] Karpiński T.M. (2020). Essential Oils of Lamiaceae Family Plants as Antifungals. Biomolecules.

[11] Chouhan S., Sharma K., Guleria S. (2017). Antimicrobial Activity of Some Essential Oils—Present Status and Future Perspectives. Medicines.

[12] Serio A., Chiarini M., Tettamanti E., Paparella A. (2010). Electronic Paramagnetic Resonance Investigation of the Activity of *Origanum vulgare* L. Essential Oil on the *Listeria monocytogenes* Membrane. Lett. Appl. Microbiol..

[13] Tian J., Ban X., Zeng H., He J., Chen Y., Wang Y. (2012). The Mechanism of Antifungal Action of Essential Oil from Dill (*Anethum graveolens* L.) on Aspergillus Flavus. PLoS ONE.

[14] Ahmad A., Khan A., Kumar P., Bhatt R.P., Manzoor N. (2011). Antifungal Activity of Coriaria Nepalensis Essential Oil by Disrupting Ergosterol Biosynthesis and Membrane Integrity against Candida. Yeast.

[15] Zeng H., Chen X., Liang J. (2015). In Vitro Antifungal Activity and Mechanism of Essential Oil from Fennel (*Foeniculum vulgare* L.) on Dermatophyte Species. J. Med. Microbiol..

[16] Pinto L., Tapia-Rodríguez M.R., Baruzzi F., Ayala-Zavala J.F. (2023). Plant Antimicrobials for Food Quality and Safety: Recent Views and Future Challenges. Foods.

[17] D’agostino M., Tesse N., Frippiat J.P., Machouart M., Debourgogne A. (2019). Essential Oils and Their Natural Active Compounds Presenting Antifungal Properties. Molecules.

[18] Nazzaro F., Fratianni F., Coppola R., De Feo V. (2017). Essential Oils and Antifungal Activity. Pharmaceuticals.

[19] Carvalho D.D.C., Alves E., Barbosa Camargos R., Ferreira Oliveira D., Soares Scolforo J.R., de Carvalho D.A., Sâmia Batista T.R. (2011). Plant Extracts to Control Alternaria Alternata in Murcott Tangor Fruits. Rev. Iberoam. Micol..

[20] Chávez-Quintal P., González-Flores T., Rodríguez-Buenfil I., Gallegos-Tintoré S. (2011). Antifungal Activity in Ethanolic Extracts of *Carica papaya* L. Cv. Maradol Leaves and Seeds. Indian. J. Microbiol..

[21] Haddouchi F., Chaouche T.M., Zaouali Y., Ksouri R., Attou A., Benmansour A. (2013). Chemical Composition and Antimicrobial Activity of the Essential Oils from Four Ruta Species Growing in Algeria. Food Chem..

[22] Oliva A., Meepagala K.M., Wedge D.E., Harries D., Hale A.L., Aliotta G., Duke S.O. (2003). Natural Fungicides from *Ruta graveolens* L. Leaves, Including a New Quinolone Alkaloid. J. Agric. Food Chem..

[23] Quiroga R., del Pilar A. (2003). Conocimiento y Uso de Las Plantas Medicinales En El Municipio de Zipacón, Cundinamarca. Bachelor’s Thesis.

[24] Colombia F.C. (2003). Manual de Fitoprotección y Analisis de Plaguicidas.

[25] Álvarez L.E.G. (2006). Cartilla Para Educación Agroecológica.

[26] Peralta-Ruiz Y., Rossi C., Grande-Tovar C.D., Chaves-López C. (2023). Green Management of Postharvest Anthracnose Caused by *Colletotrichum gloeosporioides*. J. Fungi.

[27] Landi L., Peralta-Ruiz Y., Chaves-López C., Romanazzi G. (2021). Chitosan Coating Enriched With *Ruta graveolens* L. Essential Oil Reduces Postharvest Anthracnose of Papaya (*Carica papaya* L.) and Modulates Defense-Related Gene Expression. Front. Plant Sci..

[28] Peralta-Ruiz Y., Grande Tovar C., Sinning-Mangonez A., Bermont D., Pérez Cordero A., Paparella A., Chaves-López C. (2020). *Colletotrichum gloesporioides* Inhibition Using Chitosan-*Ruta graveolens* L. Essential Oil Coatings: Studies in Vitro and in Situ on *Carica papaya* Fruit. Int. J. Food Microbiol..

[29] Molina-Hernandez J.B., Aceto A., Bucciarelli T., Paludi D., Valbonetti L., Zilli K., Scotti L., Chaves-López C. (2021). The Membrane Depolarization and Increase Intracellular Calcium Level Produced by Silver Nanoclusters Are Responsible for Bacterial Death. Sci. Rep..

[30] Tanaka M., Kanatsuka H., Ong B.-H., Tanikawa T., Uruno A., Komaru T., Koshida R., Shirato K. (2003). Cytochrome P-450 Metabolites but Not NO, PGI_2_, and H_2_O_2_ Contribute to ACh-Induced Hyperpolarization of Pressurized Canine Coronary Microvessels. Am. J. Physiol. -Heart Circ. Physiol..

[31] Vylkova S. (2017). Environmental PH Modulation by Pathogenic Fungi as a Strategy to Conquer the Host. PLoS Pathog..

[32] Patrignani F., Iucci L., Belletti N., Gardini F., Guerzoni M.E., Lanciotti R. (2008). Effects of Sub-Lethal Concentrations of Hexanal and 2-(*E*)-Hexenal on Membrane Fatty Acid Composition and Volatile Compounds of *Listeria monocytogenes*, *Staphylococcus aureus*, *Salmonella enteritidis* and *Escherichia coli*. Int. J. Food Microbiol..

[33] Oliver J.D., Colwell R.R. (1973). Extractable Lipids of Gram-Negative Marine Bacteria: Fatty-Acid Composition. Int. J. Syst. Evol. Microbiol..

[34] Nguyen H.N., Chaves-Lopez C., Oliveira R.C., Paparella A., Rodrigues D.F. (2019). Cellular and Metabolic Approaches to Investigate the Effects of Graphene and Graphene Oxide in the Fungi *Aspergillus flavus* and *Aspergillus niger*. Carbon N. Y..

[35] Ferreira P., Cardoso T., Ferreira F., Fernandes-Ferreira M., Piper P., João Sousa M. (2014). Mentha Piperita Essential Oil Induces Apoptosis in Yeast Associated with Both Cytosolic and Mitochondrial ROS-Mediated Damage. FEMS Yeast Res..

[36] Xing Y., Xu Q., Li X., Chen C., Ma L., Li S., Che Z., Lin H. (2016). Chitosan-Based Coating with Antimicrobial Agents: Preparation, Property, Mechanism, and Application Effectiveness on Fruits and Vegetables. Int. J. Polym. Sci..

[37] Chaves-Lopez C., Nguyen H.N., Oliveira R.C., Nadres E.T., Paparella A., Rodrigues D.F. (2018). A Morphological, Enzymatic and Metabolic Approach to Elucidate Apoptotic-like Cell Death in Fungi Exposed to h- and α-Molybdenum Trioxide Nanoparticles. Nanoscale.

[38] Donato R., Sacco C., Pini G., Rita A. (2020). Antifungal Activity of Different Essential Oils against *Malassezia* Pathogenic Species. J. Ethnopharmacol..

[39] Tariq S., Wani S., Rasool W., Shafi K., Ahmad M., Prabhakar A., Hussain A., Rather M.A. (2019). Microbial Pathogenesis A Comprehensive Review of the Antibacterial, Antifungal and Antiviral Potential of Essential Oils and Their Chemical Constituents against Drug- Resistant Microbial Pathogens. Microb. Pathog..

[40] Cho J., Choi H., Lee J., Kim M., Sohn H., Gun D. (2013). The Antifungal Activity and Membrane-Disruptive Action of Dioscin Extracted from *Dioscorea nipponica*. Biochim. Biophys. Acta (BBA)-Biomembr..

[41] Grande-Tovar C.D., Chaves-Lopez C., Serio A., Rossi C., Paparella A. (2018). Chitosan Coatings Enriched with Essential Oils: Effects on Fungi Involve in Fruit Decay and Mechanisms of Action. Trends Food Sci. Technol..

[42] Tian J., Wang Y., Zeng H., Li Z., Zhang P., Tessema A., Peng X. (2015). Efficacy and Possible Mechanisms of Perillaldehyde in Control of *Aspergillus niger* Causing Grape Decay. Int. J. Food Microbiol..

[43] Paul S., Dubey R.C., Maheswari D.K., Chul S. (2011). *Trachyspermum ammi* (L.) Fruit Essential Oil in Fl Uencing on Membrane Permeability and Surface Characteristics in Inhibiting Food-Borne Pathogens. Food Control.

[44] Song C., Zhang Y., Zhao Q., Chen M., Zhang Y., Gao C., Jia Z., Song S., Guan J., Shang Z. (2024). Volatile Organic Compounds Produced by *Bacillus aryabhattai* AYG1023 against *Penicillium expansum* Causing Blue Mold on the Huangguan Pear. Microbiol. Res..

[45] Badea M.L., Iconaru S.L., Groza A., Chifiriuc M.C., Beuran M., Predoi D. (2019). Peppermint Essential Oil-Doped Hydroxyapatite Nanoparticles with Antimicrobial Properties. Molecules.

[46] Raita M.S., Iconaru S.L., Groza A., Cimpeanu C., Predoi G., Ghegoiu L., Badea M.L., Chifiriuc M.C., Marutescu L., Trusca R. (2020). Multifunctional Hydroxyapatite Coated with *Arthemisia absinthium* Composites. Molecules.

[47] Herath H., Abeywickrama K. (2008). In Vitro Application of Selected Essential Oils and Their Major Components in Controlling Fungal Pathogens of Crown Rot in Embul Banana (*Musa acuminata*—AAB). Int. J. Food Sci. Technol..

[48] Tao N., OuYang Q., Jia L. (2014). Citral Inhibits Mycelial Growth of *Penicillium italicum* by a Membrane Damage Mechanism. Food Control.

[49] Chen C., Li Q., Zeng Z., Duan S., Wang W., Xu F. (2020). Efficacy and Mechanism of *Mentha haplocalyx* and *Schizonepeta tenuifolia* Essential Oils on the Inhibition of *Panax notoginseng* Pathogens. Ind. Crops Prod..

[50] Guan N., Liu L. (2020). Microbial Response to Acid Stress: Mechanisms and Applications. Appl. Microbiol. Biotechnol..

[51] Russell N.J., Evans R.I., ter Steeg P.F., Hellemons J., Verheul A., Abee T. (1995). Membranes as a Target for Stress Adaptation. Int. J. Food Microbiol..

[52] Yan D., Lin X., Qi Y., Liu H., Chen X., Liu L., Chen J. (2016). Crz1p Regulates PH Homeostasis in *Candida glabrata* by Altering Membrane Lipid Composition. Appl. Environ. Microbiol..

[53] Liu J., Zhu Y., Du G., Zhou J., Chen J. (2013). Exogenous Ergosterol Protects *Saccharomyces cerevisiae* from D-Limonene Stress. J. Appl. Microbiol..

[54] Sikkema J., de Bont J.A., Poolman B. (1995). Mechanisms of Membrane Toxicity of Hydrocarbons. Microbiol. Mol. Biol. Rev..

[55] Wang D., Wang G., Wang J., Zhai H., Xue X. (2023). Inhibitory Effect and Underlying Mechanism of Cinnamon and Clove Essential Oils on *Botryosphaeria dothidea* and *Colletotrichum gloeosporioides* Causing Rots in Postharvest Bagging-Free Apple Fruits. Front. Microbiol..

[56] Yuan T., Hua Y., Zhang D., Yang C., Lai Y., Li M., Ding S., Li S., Chen Y. (2024). Efficacy and Antifungal Mechanism of Rosemary Essential Oil against *Colletotrichum gloeosporioides*. Forests.

[57] Helal G.A., Sarhan M.M., Abu Shahla A.N.K., Abou El-Khair E.K. (2006). Effects of *Cymbopogon citratus* L. Essential Oil on the Growth, Lipid Content and Morphogenesis of *Aspergillus niger* ML2-strain. J. Basic. Microbiol..

[58] Di Pasqua R., Hoskins N., Betts G., Mauriello G. (2006). Changes in Membrane Fatty Acids Composition of Microbial Cells Induced by Addiction of Thymol, Carvacrol, Limonene, Cinnamaldehyde, and Eugenol in the Growing Media. J. Agric. Food Chem..

[59] Fozo E.M., Quivey Jr R.G. (2004). The *FabM* Gene Product of *Streptococcus mutans* Is Responsible for the Synthesis of Monounsaturated Fatty Acids and Is Necessary for Survival at Low PH. J. Bacteriol..

[60] Zhou D.R., Eid R., Boucher E., Miller K.A., Mandato C.A., Greenwood M.T. (2019). Stress Is an Agonist for the Induction of Programmed Cell Death: A Review. Biochim. Biophys. Acta (BBA)-Mol. Cell Res..

[61] Liu P., Luo L., Guo J., Liu H., Wang B., Deng B. (2010). Farnesol Induces Apoptosis and Oxidative Stress in the Fungal Pathogen *Penicillium expansum*. Micologia.

[62] Khani S., Seyedjavadi S.S., Hosseini H.M., Goudarzi M., Valadbeigi S., Khatami S., Ajdary S., Eslamifar A., Amani J., Imani Fooladi A.A. (2020). Effects of the Antifungal Peptide Skh-AMP1 Derived from *Satureja khuzistanica* on Cell Membrane Permeability, ROS Production, and Cell Morphology of Conidia and Hyphae of *Aspergillus fumigatus*. Peptides.

[63] Hlavatá L., Aguilaniu H., Pichová A., Nyström T. (2003). The Oncogenic*RAS2^val19^* Mutation Locks Respiration, Independently of PKA, in a Mode Prone to Generate ROS. EMBO J..

[64] Oliveira R.C., Carvajal-Moreno M., Mercado-Ruaro P., Rojo-Callejas F., Correa B. (2020). Essential Oils Trigger an Antifungal and Anti-Aflatoxigenic Effect on *Aspergillus flavus* via the Induction of Apoptosis-like Cell Death and Gene Regulation. Food Control.

[65] Plassard C., Fransson P. (2009). Regulation of Low-Molecular Weight Organic Acid Production in Fungi. Fungal Biol. Rev..

[66] Agnello A.C., Huguenot D., Van Hullebusch E.D., Esposito G. (2014). Enhanced Phytoremediation: A Review of Low Molecular Weight Organic Acids and Surfactants Used as Amendments. Crit. Rev. Environ. Sci. Technol..

[67] Pawar V.C., Thaker V.S. (2009). Acid Phosphatase and Invertase Activities of *Aspergillus niger*. Mycoscience.

[68] Freitas-Mesquita A.L., Meyer-Fernandes J.R. (2014). Biochemical Properties and Possible Roles of Ectophosphatase Activities in Fungi. Int. J. Mol. Sci..

[69] Lee W., Lee D.G. (2015). Fungicidal Mechanisms of the Antimicrobial Peptide Bac8c. Biochim. Biophys. Acta (BBA)-Biomembr..

[70] Bradford M.M. (1976). A Rapid and Sensitive Method for the Quantitation of Microgram Quantities of Protein Utilizing the Principle of Protein-Dye Binding. Anal. Biochem..

[71] Folch J., Lees M., Stanley G.H.S. (1957). A Simple Method for the Isolation and Purification of Total Lipides from Animal Tissues. J. Biol. Chem..

