# Supplementary material for: Antifungal Mechanism of Ruta graveolens Essential Oil: A Colombian Traditional Alternative against Anthracnose Caused by Colletotrichum gloeosporioides
Source: Molecules. 2024 Jul 26;29(15):3516. doi: 10.3390/molecules29153516 (PMC11314608; doi:10.3390/molecules29153516)
Supplement: Supplementary file 1 [file molecules-29-03516-s001.zip › molecules-3085412-supplementary.pdf]

Antifungal Mechanism of *Ruta graveolens* Essential Oil: A Colombian Traditional Alternative against Anthracnose Caused by *Colletotrichum gloeosporioides*

Yeimmy Peralta-Ruiz [1]*, Junior Bernardo Molina Hernandez [2] , Carlos David Grande-Tovar [3], Annalisa Serio [2], Luca Valbonetti [2], Clemencia Chaves-López [2]*

[1] Programa de Ingeniería Agroindustrial, Facultad de Ingeniería, Universidad del Atlántico, Carrera 30 Número 8-49, Puerto Colombia 081008, Colombia
[2] Faculty of Bioscience and Technology for Food, Agriculture and Environment, University of Teramo, Via R. Balzarini 1, 64100 Teramo, Italy
[3] Grupo de Investigación de Fotoquímica y Fotobiología, Universidad del Atlántico, Carrera 30 Número 8-49, Puerto Colombia 081008, Colombia

Authors for Correspondence:

Yeimmy Peralta-Ruiz, yeimmyperalta@mail.uniatlantico.edu.co (Y.P-R)

Clemencia Chaves- López, cchaveslopez@unite.it (C.C-L)

Supplementary material

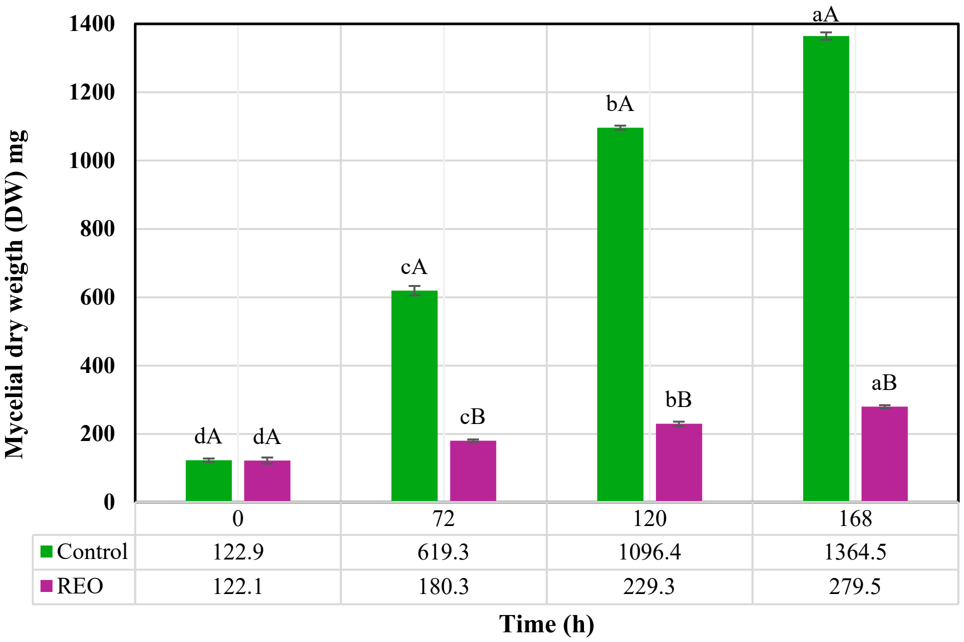

Figure S1. *C. gloeosporioides* mycelia growth reduction treated with REO

Table S1. Calibration curves, coefficients of determination (R^2), and retention time for citric, pyruvic, malic, succinic, lactic, formic, and acetic acids.

Acid	Retention time	Rage of concentration (ppm)	Calibration curve	R^2
Oxalic	5.98	30-1000	y = 68446x - 48127	0.9977
Citric	7.47	30-1000	y = 395.36x + 5797.2	0.9944
Piruvic	8.92	30-1000	y = 1244.6x - 18692	0.9986
Malic	9.07	30-1000	y = 388.52x - 17396	0.9771
Succinico	11.03	30-1000	y = 374.99x - 9884.4	0.9962
Lattic	11.61	30-1000	y = 46.595x - 794.41	0.9989
Formic	12.37	30-1000	y = 200.04x - 3387.3	0.9986
Acetic	13.4 5	30-1000	y = 271.94x + 3291.5	0.9982
